# Characterization of Bacterial Cellulose Produced by *Komagataeibacter maltaceti* P285 Isolated from Contaminated Honey Wine

**DOI:** 10.3390/microorganisms10030528

**Published:** 2022-02-28

**Authors:** Narumol Thongwai, Wirapong Futui, Nanthiwa Ladpala, Benjamat Sirichai, Anuwat Weechan, Jirapat Kanklai, Patthanasak Rungsirivanich

**Affiliations:** 1 Department of Biology, Faculty of Science, Chiang Mai University, Chiang Mai 50200, Thailand; wirapong49187@gmail.com (W.F.); nanthiwa.l.14@gmail.com (N.L.); kaebenjamat@gmail.com (B.S.); anuwat.aw80@gmail.com (A.W.); jirapat18101987@gmail.com (J.K.); 2 Research Center in Bioresources for Agriculture, Industry and Medicine, Chiang Mai University, Chiang Mai 50200, Thailand; 3 Graduate School, Chiang Mai University, Chiang Mai 50200, Thailand

**Keywords:** *Acetobacter xylinum*, biopolymer, honey, mead, optimization, sugarcane, *Novacetimonas*

## Abstract

Bacterial cellulose (BC), a biopolymer, is synthesized by BC-producing bacteria. Almost all producing strains are classified in the family *Acetobacteraceae*. In this study, bacterial strain P285 was isolated from contaminated honey wine in a honey factory in northern Thailand. Based on 16S rRNA gene sequence identification, the strain P285 revealed 99.8% identity with *Komagataeibacter maltaceti* LMG 1529 ^T^. *K. maltaceti* P285 produced the maximum BC production at 20–30 °C and an initial media pH of 9.0. The highest BC production in modified mineral salt medium (MSM) was exhibited when glucose (16%, *w*/*v*) and yeast extract (3.2%, *w*/*v*) were applied as carbon and nitrogen sources, respectively. When sugarcane (8–16%, *w*/*v*) or honey (ratio of honey to water = 1: 4) supplemented with yeast extract was used, the BC production was greater. The characterization of BC synthesized by *K. maltaceti* P285 was undertaken using scanning electron microscopy (SEM) and Fourier transform infrared (FTIR) spectrometry. Meanwhile, X-ray diffraction results confirmed the presence of crystalline cellulose (2θ = 18.330, 21.390 and 22.640°). The maximum temperature of BC degradation was observed at 314 °C. Tensile properties analysis of hydrated and dried BC showed breaking strength of 1.49 and 0.66 MPa, respectively. These results demonstrated that *K. maltaceti* P285 has a high potential for BC production especially when grown in high initial media pH. Therefore, the strain would be suitable as an agent to make BC, the value-added product in the related factories.

## 1. Introduction

*Komagataeibacter maltaceti* (formerly *Gluconacetobacter maltaceti*), a non-motile Gram-negative bacterium in the family *Acetobacteraceae*, was first described as a novel species by Slapšak et al. [1] isolated from malt vinegar. The genus *Komagataeibacter* has been classified as acetic acid bacteria (AAB). Some species exhibits bacterial cellulose (BC) production ability [2,3].

BC is a natural polymer synthesized by many species in the *Acetobacteraceae* family such as *K. hansenii*, *K. europaeus*, *K. maltaceti*, *K. melomenusus*, *K. rhaeticus* and *K. xylinus* [4,5,6,7,8]. Besides, certain species of genera *Agrobacterium*, *Burkholderia*, *Dickeya*, *Erwinia*, *Escherichia*, *Pseudomonas*, *Rhizobium* and *Salmonella* has also been described to produce BC [9]. The BC-producing bacteria can be isolated from environments (e.g., soil and plants), animals (e.g., insects), humans, foods, beverages (e.g., kombucha tea and vinegar), vegetables, fruit juices and decaying fruits [10,11].

BC produced by bacteria has been considered as having high purity and water absorption ability, It is non-toxic, has a low density and retaining capacity, and provides biocompatibility and biodegradability [8,12]. BC is synthesized in the periplasmic space of a bacterial cell from nucleotide-activated glucose using an enzymatic complex (cellulose synthase) [9] and then extruded out through the pores of cell membrane as D-glucose linear chains. The glucose units are linked with β-1,4-glycosidic bonds. Subsequently, the microfibrils are formed by hydrogen bonding between chains. These ribbon-shaped fibrils create a structure of network and extend on the surface area [5,12,13]. BC has long been used as foods and food related products [14]. Additionally, BC can be applied in the cosmetic industry as a stabilizer and component of facial mask and scrub [15]. In biomedicine field, BC has been used as a biomedical material for recuperation of wound on skin by moist environment preservation together with the retention of exudate [16]. Moreover, BC can also be used as an artificial skin and blood vessel as well as carriers for drug delivery [17].

Cane (*Saccharum officinarum*) is a tropical perennial plant which mostly cultivated for its sugar contents [18]. Sugarcane (brown granulated sugar) is generated after sugar agroindustrial process. Sugarcane mainly contains sugars in particular sucrose. Moreover, sugarcane also comprises of other sugars (i.e., glucose and fructose), amino acids, organic acids and inorganics (e.g., SiO_2_, K_2_O, P_2_O_3_ and Fe_2_O_3_) [18,19]. Sugarcane has generally been used as an ingredient in food and dessert. Besides, it is also used for ethanol production in alternative energy applications [20]. Honey is a natural food, mostly consisting of sugar (e.g., glucose, fructose, sucrose, maltose, trehalose, rhamnose and raffinose) [21] and other compositions such as amino acids (e.g., proline, glutamic acid, glutamine, glycine, lysine and tryptophan) [22], organic acids (e.g., citric, formic, gluconic, lactic, tartaric and propionic acids) [23], vitamins (e.g., thiamine, riboflavin, nicotinic acid, pyridoxine and vitamin C) [24] and minerals (potassium, calcium, phosphorus, manganese and zinc) [25]. Furthermore, honey has plenty of antioxidants such as flavonoids and phenolic acids [26]. Therefore, honey is popularly used as an ingredient in food, medical and pharmaceutical industries due to its properties. Both sugarcane and honey are abundant in Thailand.

The high cost of fermentation media is generally a problem in industries [27]. This study aimed to isolate cellulose producing bacteria from contaminated honey wine, to evaluate the influences of factors including pH, temperature, carbon and nitrogen sources for BC production. Honey and sugarcane were evaluated for their potential to be raw materials for BC production by the isolated *K. maltaceti* in order to apply in food and food related or non-food factories in the future. The BC obtained was structurally characterized using scanning electron microscopy (SEM), Fourier transform infrared (FTIR) spectroscopy, X-ray diffraction (XRD), differential scanning calorimetry (DSC) and mechanical testing.

## 2. Materials and Methods

### 2.1. Reference Bacterial Strain and Growth Conditions

*Komagataeibacter nataicola* TISTR 975 (formerly identified as *Acetobacter xylinum* TISTR 975) purchased from the Thailand Institute of Scientific and Technological Research, Pathum Thani, Thailand was used as a control. *K. nataicola* TISTR 975 was grown in Hestrin-Schramm (HS) broth (20 g/L glucose, 2 g/L peptone, 5 g/L yeast extract, 2.7 g/L Na_2_HPO_4_, 1.2 g/L citric acid monohydrate, pH 6.0) at 30 °C for 48–72 h, as previous described by Hestrin and Schrammn [28].

### 2.2. Bacterial Isolation

Pellicle on the surface of contaminated honey wine obtained from Bee Products Industry Co., Ltd. (Chiang Mai, Thailand) was collected, and BC (Nata de coco) was bought from a market in Pathum Thani province, Thailand. Approximately 1 g of the cellulose pellicle was soaked in 5 mL of HS broth prior to shaking at room temperature for 5 min. The suspension was spread onto HS agar and incubated at 30 °C for 48 h. The pure bacterial isolates were kept at −20 °C in HS broth supplemented with 20% (*v*/*v*) of glycerol. All media used for isolation were Merck™ (Darmstadt, Germany).

### 2.3. Morphological, Biochemical and Physiological Characterization of the Selected Bacterial Isolate

Bacterial isolates were characterized by the observation of colony morphology, Gram reaction, and string and motility tests. Cell morphology was investigated using SEM (JSM-IT300, JEOL Ltd., Tokyo, Japan) according to the protocol of Arroyo et al. [29]. For biochemical identification, an evaluation of various tests was assessed, consisting of catalase, indole, lysine decarboxylase, lysine deaminase and cytochrome oxidase production, methyl red and Voges-Proskauer, nitrate reduction, citrate, triple sugar iron, gelatinase, urease and carbohydrate fermentation (including galactose, glucose, lactose, maltose, mannitol, mannose, rhamnose and sucrose) tests.

### 2.4. Genomic DNA Extraction and 16S rRNA Gene Amplification

The selected bacterial isolates were identified using 16S rRNA gene analysis. The genomic DNA was extracted according to the method of Pitcher et al. [30] with slight modifications. The polymerase chain reaction (PCR) was used for amplifying the 16S rRNA gene with universal bacterial primers, 27F (5′-AGA GTT TGA TCM TGG CTC AG-3′) and 1492R (5′-TAC GGY TAC CTT GTT ACG ACT T-3′) [31]. The amplification method was accomplished according to the procedure described by Rungsirivanich et al. [32] using electrophoretic gel system (EC320, Minicell Primo, San Diego, CA, USA). The PCR products were purified and sequenced by a DNA sequencing service (First BASE Laboratories Sdn Bhd., Selangor, Malaysia). The 16S rRNA gene sequences were compared with other gene sequences by GenBank and EzBioCloud databases. The sequences were cleaned and assembled using FinchTV version 1.4 and BioEdit version 7.2.5 softwares [33]. The phylogenetic tree was constructed using MRGA 7.0 software [34] based on a neighbor-joining method [35]. Amplicon sequence was deposited in the GenBank under accession number MN559523 and OL872255.

### 2.5. Preparation of Inoculum

The selected bacterial isolates and *K. nataicola* TISRT 975 were grown on HS agar and incubated at 30 °C for 48 h. Bacterial cells were resuspended in 0.85% (*w*/*v*) NaCl and adjusted its turbidity equivalent to 1.0 at OD_600_ (approximately 1 × 10^8^ CFU/mL). Each resuspended culture was designated as the starting culture for all experiments with 5% (*v*/*v*) inoculum size.

### 2.6. Effect of Temperature and Initial Media pH on BC Production

To evaluate the optimum temperature and initial media pH for BC production, the starting culture of *K. maltaceti* P285 and *K. pomaceti* O277 was inoculated (5%, *v*/*v*) into 50 mL of HS broth, pH 6.0, in 125 mL Erlenmeyer flasks. The flasks were incubated in undisturbed state at various temperatures (20, 25, 30, 35, 37 and 40 °C) for 7 days. The *K. nataicola* TISRT 975 was used as a control. Meanwhile, the effect of the initial media pH on BC production by the strains P285, O277 and TISTR 975 were performed by adjusting the HS broth pH to 3, 4, 5, 6, 7, 8, 9, 10, 11 and 12 using 1 M HCl or NaOH. The HS broth with initial pH 6.0 was used as a positive control. All cultures were incubated at 30 °C for 7 days. Three independent experiments were conducted. After cultivation, the BC pellicles were harvested and cleaned as described in Section 2.10, and final media pH were measured.

### 2.7. Effect of Carbon and Nitrogen Sources on BC Production

To study the effect of carbon sources on BC production, the starting culture was inoculated (5%, *v*/*v*) into 50 mL of modified mineral salt medium (MSM; 2 g/L NaNO_3_, 1.2 g/L K_2_HPO_4_, 0.5 g/L KH_2_PO_4_, 0.14 g/L MgSO_4_ and 0.01 g/L Fe(SO_4_)_3_; [36]) with four different carbon sources including citric acid monohydrate, ethanol, glucose and sucrose. The various concentration of carbon sources (1, 2, 4, 8, 16 and 32% (*w*/*v*) or (*v*/*v*) for ethanol) were supplied. The MSM broth with 2% (*w*/*v*) glucose and MSM broth without carbon source were used as positive and negative controls. Meanwhile, the effect of nitrogen sources were assessed using modified MSM (20 g/L glucose, 1.2 g/L K_2_HPO_4_, 0.5 g/L KH_2_PO_4_, 0.14 g/L MgSO_4_ and 0.01 g/L Fe(SO_4_)_3_) by varying nitrogen sources (peptone, yeast extract, (NH_4_)_2_HPO_4_ and NaNO_3_). The different nitrogen source concentrations (0, 0.1, 0.2, 0.4, 0.8, 1.6 and 3.2% (*w*/*v*)) were evaluated. The MSM broth with 0.2% (*w*/*v*) NaNO_3_ and MSM broth without nitrogen source were designed as positive and negative controls. All cultures were incubated at 30 °C for 7 days. The experiments were performed as three independent replicates. After cultivation, the cellulose was harvested and weighted as mentioned in the Section 2.10. The final media pH values were investigated.

### 2.8. Effect of Sugarcane and Honey on BC Production by the Selected Bacterial Isolates

To evaluate the effect of sugarcane and honey on BC production by selected bacterial isolates, the starting cultures of *K. maltaceti* P285, *K. pomaceti* O277 and *K. nataicola* TISTR 975 (control) were used to inoculate (5%, *v*/*v*) into 50 mL of various concentrations of sugarcane (Mitr Phol, Chaiyaphum, Thailand; 2, 4, 8, 16 and 32%, *w*/*v*) and various ratios of water diluted honey (Bee Products Industry Co., Ltd., Thailand; honey: water of 1:1, 1:2, 1:4, 1:8 and 1:16) supplemented with 0.2% (*w*/*v*) yeast extract. All experiments were adjusted the pH to 6.0 using 1 m HCl or NaOH. The HS broth was used as a control. The experiments were conducted in three independent treatments. After static cultivation at 30 °C for 7 days, the BC pellicles produced on the media surface were harvested and cleaned as described in the Section 2.10. The final pH value of culture broths was ultimately measured.

### 2.9. Sugarcane and Honey Optimization of BC Production by Selected Bacterial Strains

According to data acquired, the presence of sugarcane (8%, *w*/*v*) or honey solution (1:4 of honey: water ratio), yeast extract (3.2%, *w*/*v*) and initial media pH 9.0 influenced the BC production of *K. maltaceti* P285. A 5% (*v*/*v*) of each starter culture was inoculated into new formulas of sugarcane (S1 and S2) and honey (H1 and H2) solutions before incubation at 30 °C for 7 days. The component of solutions is represented in Table 1. The HS broth and the solutions of sugarcane (8%, *w*/*v*) and honey (1:4 of honey: water ratio) supplemented with 0.2% (*w*/*v*) yeast extract, pH 6.0 were used as controls. The experiments were performed as three independent replicates. After cultivation, the cellulose was harvested and cleaned as described below. The final pH of solutions was evaluated.

### 2.10. Harvesting and Weighing of BC

After cultivation, the BC pellicles were soaked in distilled water for 2 h prior to washing with distilled water at least three times to remove bacterial cell, acid-base and residual media, sugarcane or honey solutions. Cleaned BC was subsequent dried at 60 °C for 24 h before measurement of dry weight as previously described by Costa et al. [12] with slight modifications.

### 2.11. Physical Property Determination of BC

#### 2.11.1. Scanning Electron Microscopy

The dried BC sheet was mounted on a copper stub by double adhesive carbon conductive tape prior to coating with gold for 30 s. The characteristic of BC was examined by a scanning electron microscopy (EFI Quanta 200 3D, Eugene, OR, USA) operating at 15.0 kV at room temperature.

#### 2.11.2. Fourier Transform Infrared (FTIR) Spectroscopy

The BC FTIR spectra were analyzed by modifications of the protocol explained by Atykyan et al. [37]. The dried BC pellicle sheet was cut to a size of 5 mm × 5 mm, before the transmission measurements using FTIR spectrometer (Nicolet 6700, Thermo Scientific^TM^, Waltham, MA, USA). The spectra were recorded in the spectral region of 4000–400 cm^−1^ range with a resolution of 6 cm^−1^.

#### 2.11.3. X-ray Diffractometry

The X-ray diffraction analysis was conducted according to the method of Bandyopadhyay et al. [38] with modifications. The X-ray diffraction patterns of BC sheets were measured by a diffractometer (Rigaku, MiniFlex II, Tokyo, Japan) operating at 40 kV, 40 mA. The diffractograms were taken from 0 to 60° in a 2θ scale with a step size of 0.02°.

#### 2.11.4. Differential Scanning Calorimetry (DSC) Analysis

The thermal property of BC was analyzed using a differential scanning calorimeter (DSC 1 STAR System Mettler Toledo^TM^, Columbus, OH, USA). Approximately 5–10 mg of dried BC was heated from 20 to 500 °C with a heating rate of 10 °C/min. The empty aluminum pan was used as a reference [39].

#### 2.11.5. Tensile Strength Determination

Tensile testing was performed according to the protocol of Costa et al. [12] with modifications. The dried (60 °C for 24 h) and hydrated (60 °C for 4 h) BC sheets were cut into rectangular strips, 10 mm × 70 mm. Tensile testing was done using a tensometer (Hounsfield-H10KS, CA, USA) at room temperature with a speed of 10.0 mm/min. Young’s modulus of elasticity (MPa) and elongation at break (%) were investigated.

### 2.12. Statistical Analysis

A one-way analysis of variance (ANOVA) test was used for calculating the statistical significance of pH, temperature, carbon and nitrogen sources effects as well as the influence of sugarcane and honey on BC production using SPSS 22.0 software and Duncan’s multiple range tests. A *p*-value less than 0.05 (*p* < 0.05) was considered statistical significance.

## 3. Results

### 3.1. Bacterial Isolation and Identification

Two isolates, namely P285 and O277, were isolated from contaminated honey wine and Nata de coco samples with cellulose sheet. The strains P285 and O277 were Gram negative short rod arranged in pairs and single, and non-motile. For biochemical characterization, the strains P285 and O277 were shown positive results for catalase, lysine decarboxylase, methyl red and string tests. In contrast, the negative results were represented for cytochrome oxidase, gelatinase, indole, lysine deaminase, Voges-Proskauer, nitrate reduction and urease tests in both P285 and O277 strains. Moreover, the isolates P285 and O277 produced acid from glucose and galactose, and weak acid from maltose and sucrose. The biochemical characteristics of isolates P285 and O277 are presented in Table 2. *K. nataicola* TISTR 975 was used as a control.

**Figure 1:**
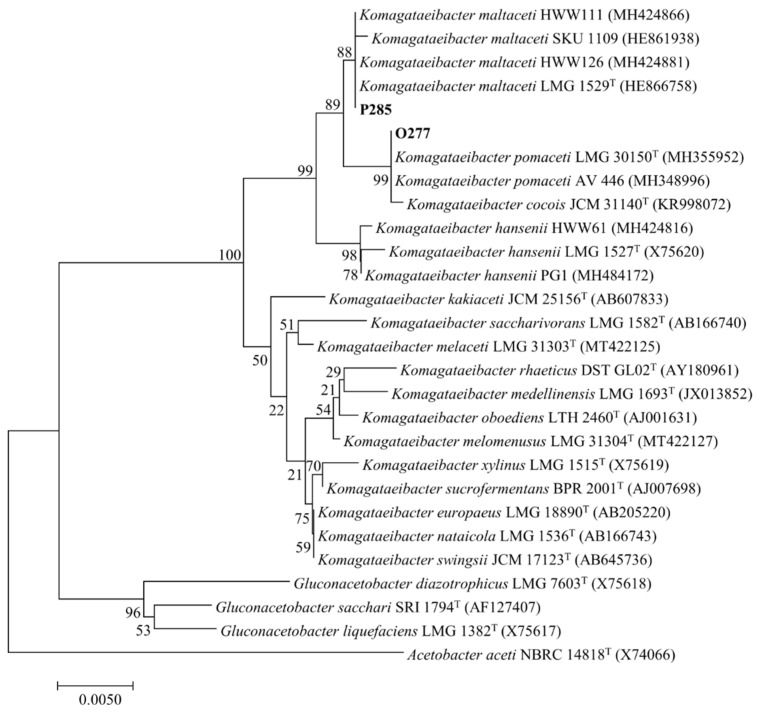
Phylogenetic relationships of the strains P285 and O277 (bold), some species of the genus *Komagataeibacter* and *Gluconacetobacter*, and related taxa based on 16S rRNA gene sequence analysis. The branching pattern was generated by the neighbour-joining method. Bootstrap values (expressed as percentages of 1000 replications). Bar, 0.005 substitutions per nucleotide position. *Acetobacter aceti* NBRC 14818 ^T^ (GenBank accession number X74066) is demonstrated as outgroup sequence.

For molecular identification, the 16S rRNA gene nucleotide sequences of the strains P285 (1306 bps, GenBank accession number MN559523) and O277 (1375 bps, GenBank accession number OL872255), were investigated. The phylogenetic tree of relationships was analyzed using the 28 aligned sequences. The strain P285 shared 99.8 and 99.1% similarity to *K. maltaceti* LMG 1529 ^T^ and *K. hansenii* LMG 1527 ^T^, respectively (nucleotide substitution 3 and 12 positions, respectively). Meanwhile, the strain O277 exhibited the 16S rRNA gene identity of 99.7 and 99.3% with *K. pomaceti* LMG 30150^T^ and *K. maltaceti* LMG 1529^T^, respectively (nucleotide substitution 4 and 9 positions, respectively). The phylogenetic tree supported by bootstrap values of 1000 replications using the neighbour-joining method is presented in Figure 1.

### 3.2. Effect of Temperatures and Initial Media pH on BC Production by Selected Bacterial Isolates

**Figure 2:**
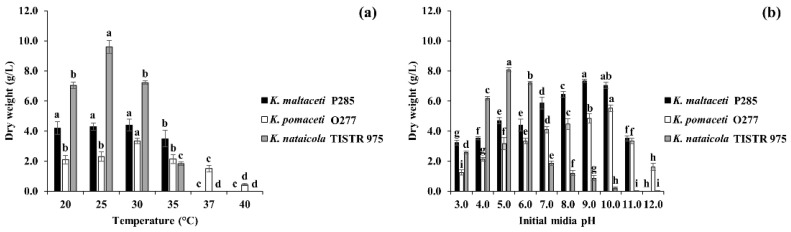
Effect of temperature (**a**); and initial media pH (**b**) on bacterial cellulose production by *K. maltaceti* P285 and *K. pomaceti* O277 cultured in HS medium. The *K. nataicola* TISTR 975 was used as control. Data expressed mean ± standard deviation (*n* = 3). The difference letters were considered statistically significant (*p* < 0.05) for each bacterial strain.

To study the effect of temperatures and initial media pH on BC production, *K. maltaceti* P285 and *K. pomaceti* O277 (assigned as a commercial strain) were inoculated into HS broth prior to incubation at different temperatures (20, 25, 30, 35, 37 and 40 °C) for 7 days or cultivated in HS broth with various media pH (3, 4, 5, 6, 7, 8, 9, 10, 11 and 12) before incubation at 30 °C for 7 days. The *K. nataicola* TISTR 975 was used as a control. The production of BC by the strain P285 cultivated in HS broth and incubated at 20, 25 and 30 °C for 7 days were 4.2 ± 0.0, 4.3 ± 0.1 and 4.4 ± 0.5 g/L, respectively while the strain O277 demonstrated BC production of 3.3 ± 0.2, 2.3 ± 0.3 and 2.1 ± 0.3 g/L, a significant (*p* < 0.05) when compared with strain P285. The *K. nataicola* TISTR 975 was shown to produce BC of 7.2 ± 0.1, 9.6 ± 0.4 and 7.0 ± 0.2 g/L at 20, 25 and 30 °C, respectively. The BC production yield of strains P285 and TISTR 975 were significantly decreased after incubation at 35 °C and were unable to produce BC at 37 and 40 °C. Interestingly, the *K. pomaceti* O277 was shown to elicit BC production at 37 °C (1.5 g/L) and 40 °C (0.4 g/L). The effect of temperatures on BC production is presented in Figure 2a. The final media pH was exhibited between 3.16 and 5.98 (Supplementary Materials Figure S1).

For the initial media pH effect, the *K. maltaceti* P285, *K. pomaceti* O277 and *K. nataicola* TISTR 975 showed the highest BC production yield of 7.3 ± 0.1, 5.5 ± 0.2 and 8.1 ± 0.1 g/L with the initial media pH of 9.0, 10.0 and 5.0, respectively, a significant difference (*p* < 0.05). The BC production of strains P285 and O277 were gently decreased by 11.6–56.0% and 12.3–77.4%, respectively when the initial media pH was lower. At the initial media pH 11.0 and 12.0, the strains P285 and O277 significantly showed the reduction of BC production (*p* < 0.05). On the other hand, the BC production yield of *K. nataicola* TISTR 975 was significantly decreased when cultured in HS broth with initial media pH of 3.0–4.0 and 6.0–11.0, and it was unable to produce BC at initial pH of 12.0. The final pH was 2.54–6.83 after cultivation for 7 days (Figure 2b and Supplementary Materials Figure S1).

### 3.3. Effect of Carbon and Nitrogen Sources on BC Production

**Figure 3:**
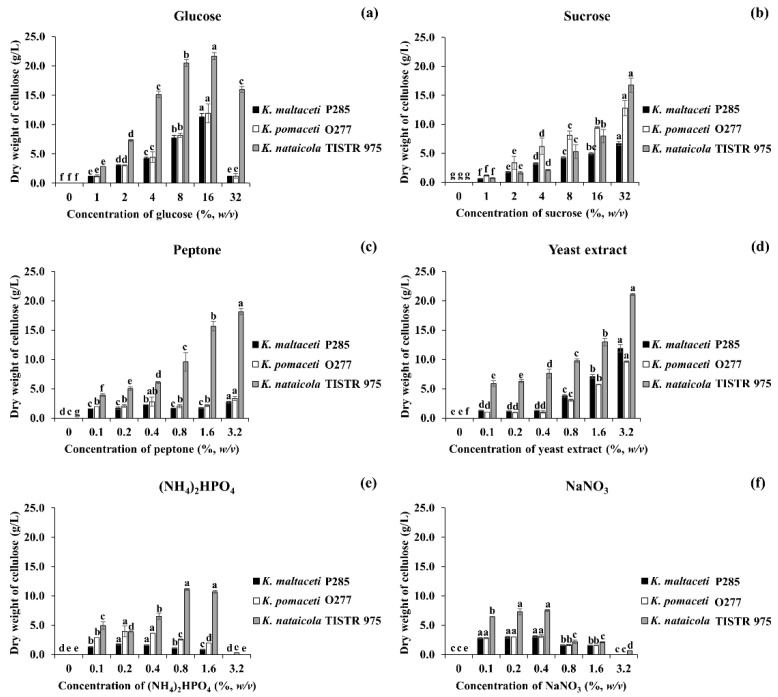
Dry mass of bacterial cellulose obtained from modified MSM with various concentrations of carbon, glucose (**a**); sucrose (**b**); and nitrogen sources, peptone (**c**); yeast extract (**d**); (NH_4_)_2_HPO_4_ (**e**)_;_ and NaNO_3_ (**f**) produced by *K. maltaceti* P285 and *K. pomaceti* O277. The *K. nataicola* TISTR 975 was used as a control. Data expressed mean ± standard deviation of three independent experiments. The difference letters were considered statistically significant (*p* < 0.05) for each bacterial strain.

To investigate the influence of carbon and nitrogen sources on BC production by *K. maltaceti* P285 and *K. pomaceti* O277, all strains were cultivated in modified MSM with various carbon or nitrogen sources and incubated at 30 °C for 7 days. The cultivation in modified MSM broth supplemented with 16% (*w*/*v*) glucose or 32% (*w*/*v*) sucrose as a carbon source was shown to exhibit the highest BC production significantly (*p* < 0.05), 11.3–14.3 and 6.7–16.7 g/L, respectively (Figure 3a,b). Conversely, the strains P285 and O277 were unable to produce BC in modified MSM broth containing citric acid or ethanol as a carbon source, whereas *K. nataicola* TISTR 975 presented BC production between 0.1 and 0.9 g/L (Supplementary Materials Table S1). The final media pH is presented in Supplementary Materials Figure S2.

The effect of nitrogen source on BC production by the selected bacterial strains was conducted using modified MSM broth with different concentrations of nitrogen sources including peptone, yeast extract, (NH_4_)_2_HPO_4_ and NaNO_3._ All cultures grown in modified MSM broth supplemented with 3.2% (*w*/*v*) peptone or yeast extract as a nitrogen source displayed the highest BC production, 3.3–18.1 and 9.6–21.1 g/L, respectively (*p* < 0.05; Figure 3c,d). Additionally, the strains P285 and O277 demonstrated to elicit the highest BC production when cultured in modified MSM broth containing 0.2 or 0.4% (*w*/*v*) (NH_4_)_2_HPO_4_ (*p* < 0.05). Likewise, all cultures demonstrated the highest production of BC when they were cultivated in modified MSM broth comprising 0.2 or 0.4% (*w*/*v*) NaNO_3_ (3.0–7.5 g/L). Simultaneously, the strain TISTR 975 grown in MSM broth containing 0.8 or 1.6% (*w*/*v*) (NH_4_)_2_HPO_4_ significantly (*p* < 0.05) represented the highest BC production, 10.7 and 11.1 g/L, respectively (Figure 3e,f and Supplementary Materials Table S1). The obtained results suggested that the cultivation in modified MSM broth with either peptone or yeast extract as a nitrogen source expressed a higher BC production when the nitrogen source concentration was higher, which opposed to NaNO_3_ and (NH_4_)_2_HPO_4_. The final media pH after cultivation was shown to be from 2.48 to 6.51 (Supplementary Materials Figure S3).

### 3.4. BC Production Using Sugarcane and Honey by Strains P285 and O277

**Figure 4:**
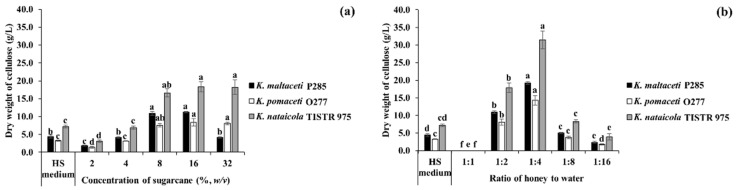
The influence of sugarcane (**a**); and honey (**b**) on bacterial cellulose production by *K. maltaceti* P285 and *K. pomaceti* O277. The *K. nataicola* TISTR 975 was used as control. Data expressed mean ± standard deviation (*n* = 3). The difference letters were considered statistically significant (*p* < 0.05) for each bacterial strain.

**Figure 5:**
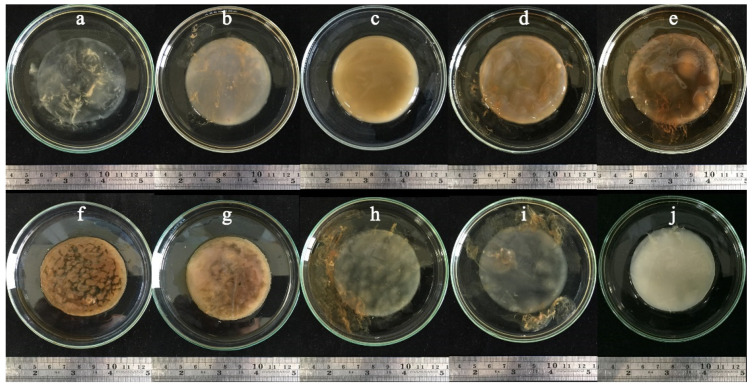
The bacterial cellulose pellicles produced by *Komagataeibacter maltaceti* P285 cultured sugarcane at the concentration of 2 (**a**); 4 (**b**); 8 (**c**); 16 (**d**); and 32 (**e**) % (*w*/*v*), honey solution at the ration of honey to water as 1:2 (**f**); 1:4 (**g**); 1:8 (**h**); and 1:16 (**i**); and HS medium (**j**). All cultures were incubated at 30 °C for 7 days.

To evaluate the potential of sugarcane and honey on BC production by selected strains. *K. maltaceti* P285 and *K. pomaceti* O277 were cultivated in various concentrations of sugarcane and honey solutions, and HS broth (control) at 30 °C for 7 days. The *K. nataicola* TISTR 975 was used as a control. Among these, the highest BC production (7.6–18.4 g/L) was insignificantly (*p* > 0.05) demonstrated in 8–16% (*w*/*v*) sugarcane, whereas a significant (*p* < 0.05) BC production (1.9–6.9 g/L) was found in the lower concentration of sugarcane (2–4%, *w*/*v*) as well as HS broth, 3.3–7.2 g/L. Furthermore, *K. nataicola* TISTR 975 was shown to produce higher amount of BC than that of the strain P285 and O277 (*p* < 0.05) (Figure 4a and Supplementary Materials Figure S4). When all strains were cultivated in honey solutions, 1:4 of honey: water ratio was shown to significantly (*p* < 0.05) elicit the highest BC production, 14.3–31.5 g/L. Meanwhile, the cultivation of *K. maltaceti* P285 and *K. pomaceti* O277 and *K. nataicola* TISTR 975 in 1:8 of honey: water ratio (3.8–8.3 g/L) presented a non-significance (*p* > 0.05) when compared to HS broth, 3.3–7.2 g/L. Noteworthy, the BC production of all strains was not observed in 1:1 of honey: water ratio (Figure 4b and Supplementary Materials Figure S4). The results indicated that sugarcane and honey solutions represented great sources for BC production. The BC pellicles produced by *K. maltaceti* P285 grown in sugarcane and honey solutions are showed in Figure 5a–j.

### 3.5. BC Production Optimization of Sugarcane and Honey by Selected Bacterial Strains

**Figure 6:**
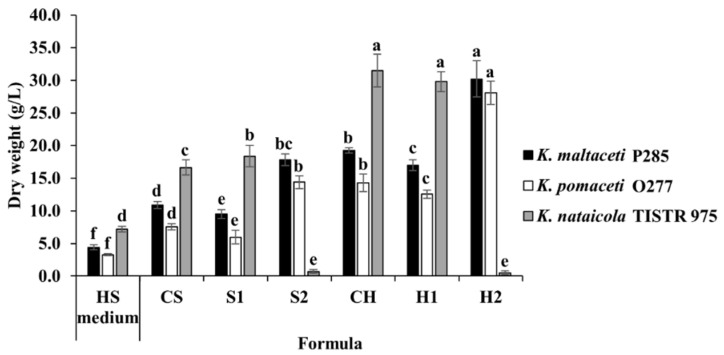
BC production of *K. maltaceti* P285 and *K. pomaceti* O277 in modified sugarcane (S1 and S2) and honey (H1 and H2) solutions. The HS broth and the solutions of sugarcane (8%, *w*/*v*; CS) and honey (1:4 of honey: water ratio; CH) supplemented with 0.2% (*w*/*v*) yeast extract, pH 6.0 were used as controls. The *K. nataicola* TISTR 975 was used as control. Data expressed mean ± standard deviation (*n* = 3). The difference letters were considered statistically significant (*p* < 0.05) for each bacterial strain.

Based on the data obtained, the presence of sugarcane (8%, *w*/*v*) or honey solution (1:4 of honey: water ratio), yeast extract (3.2%, *w*/*v*) and initial media pH 9.0 allowed the highest BC production of *K. maltaceti* P285. Four novel formulas of sugarcane and honey solutions were constructed (Table 1). The modified honey solution H2 (Honey (1:4 of honey: water ratio), yeast extract (3.2%, *w*/*v*), pH 9.0) was shown to elicit the highest BC production of *K. maltaceti* P285 and *K. pomaceti* O277, 30.2 and 28.1 g/L, respectively, which opposed to the cultivation by *K. nataicola* TISTR 975 (0.5 g/L), a significant difference (*p* < 0.05). Conversely, the maximum BC production by *K. nataicola* TISTR 975 was showed in both sugarcane and honey solutions with initial media pH 5–6 (16.6–31.5 g/L) (Figure 6). The obtained results consistent with the influence of pH on BC production which the highest BC yield was exhibited at pH 9.0 for *K. maltaceti* P285 and pH 5.0 for *K. nataicola* TISTR 975. The final pHs were 2.30–5.80 (Supplementary Materials Figure S5).

### 3.6. Scanning Electron Microscopy (SEM)

**Figure 7:**
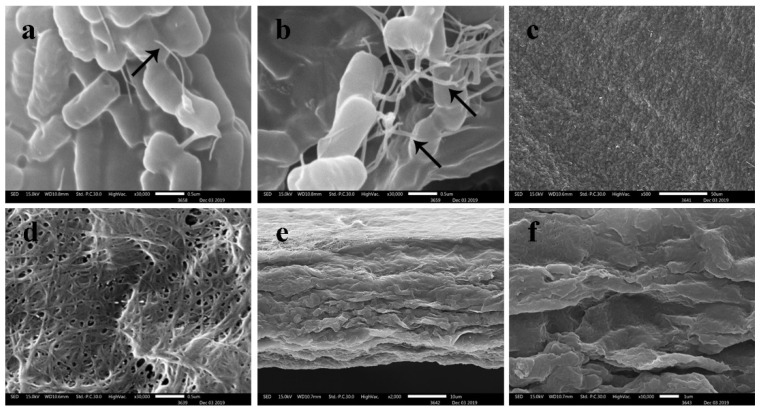
The SEM micrographs of *K. maltaceti* P285; (**a**,**b**) the morphological characteristic shows rod-shaped bacterium at magnification 30,000× and the arrows indicate bacterial cellulose formation. (**c**,**d**) bacterial cellulose surface at magnification 500× and 30,000×, respectively; (**e**,**f**) bacterial cellulose margin at magnification 2000× and 10,000×, respectively. The bacterial cellulose pellicle received from *K. maltaceti* P285 cultivated in HS medium at 30 °C for 21 days (**c**–**f**).

The structure of BC membrane produced by *K. maltaceti* P285 was investigated by SEM analysis (Figure 7). The results exposed that BC synthesized by *K. maltaceti* P285 from the cultivation at 30 °C for 21 days in HS medium was shown to comprise of fibrils which were interwoven into a reticular or network structure with porous size approximately 50–250 nm (Figure 7a–d). In addition, its margin of BC membranes displayed around 20 µm (Figure 7e,f).

### 3.7. Fourier Transform Infrared (FTIR) Spectroscopy

**Figure 8:**
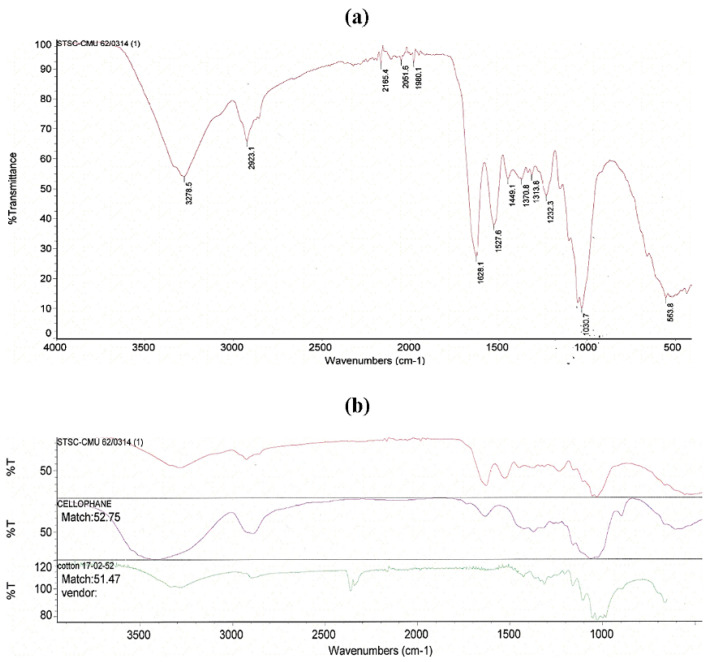
FTIR spectrum of bacterial cellulose obtained from *K. maltaceti* P285 cultivation in HS medium at 30 °C for 21 days (**a**); when compared with database (**b**).

The FTIR spectrum of BC obtained by *K. maltaceti* P285 grown in HS medium at 30 °C for 21 days is represented in Figure 8a. The FTIR spectrum pattern showed characteristic absorption patterns corresponding to the specific functional groups of α-cellulose [40,41]. The peaks at 3278.5 and 2923.1 cm^−1^ are related to −OH and C-H groups stretching, respectively. The peak at 1628.1 cm^−1^ is assigned as H_2_O absorbed. The peak appears at 1449.1 cm^−1^ may be due to CH_2_ bending vibration while the peak at around 1370.8 cm^−1^ displayed a bending of OH groups. The peak at 1030.7 cm^−1^ is considered as C-O-C pyranose ring skeletal vibration. In addition, the results indicated that the BC FTIR spectrum obtained by bacterial cultivation expressed the chemical structure corresponding to cellophane and cotton (Figure 8b).

### 3.8. X-ray Diffraction Analysis

**Figure 9:**
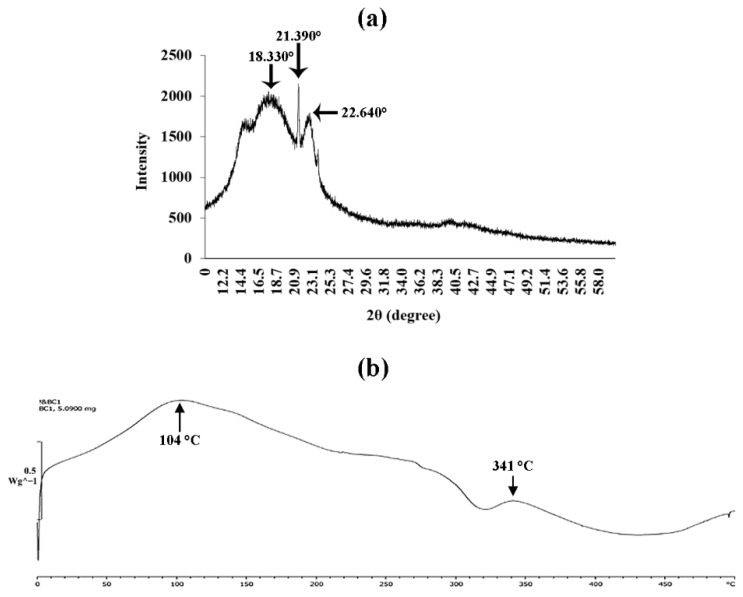
X-ray diffraction diagram (**a**); and DSC spectrum (**b**) of bacterial cellulose obtained from *K. maltaceti* P285 cultivation in HS medium and incubation at 30 °C for 21 days.

The crystalline structure and change in crystallinity of BC produced by the strain P285 were investigated by X-ray diffraction. The peaks at 2θ angles of dried BC obtained after incubation for 21 days in HS medium were 18.330, 21.390 and 22.640°. The X-ray diffraction diagram of BC is displayed in Figure 9a.

### 3.9. Differential Scanning Calorimetry (DSC) Analysis

The DSC was investigated to elucidate the property of energy consumption of BC. The peak at 104 °C occurred the transformation which led to crystalline melting of BC. Meanwhile, the maximum decomposition temperature of BC membranes occurred at 341 °C (Figure 9b).

### 3.10. Tensile Strength Analysis

**Figure 10:**
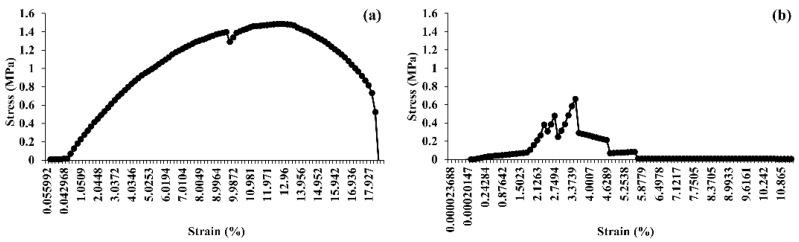
Mechanical property of hydrated (**a**); and dried (**b**) bacterial cellulose pellicles produced by *K. maltaceti* P285 after incubation at 30 °C for 21 days in HS medium.

The mechanical properties of hydrated (60 °C for 4 h) and dried (60 °C for 24 h) BC pellicles produced by *K. maltaceti* P285 were performed by tensile assay. The hydrated BC pellicles demonstrated a higher Young’s modulus than dried BC films, 1.49 (Figure 10a) and 0.66 (Figure 10b) MPa, respectively. Meanwhile, the elongation at break was 12.56 and 3.50% for hydrated and dry pellicles of BC, respectively.

## 4. Discussion

*K. xylinus* is a well-known BC producer for a long period of time. Presently, BC production has also been found in other species e.g., *K. hansenii*, *K. maltaceti*, *K. medellinensis*, *K. melomenusus*, *K. rhaeticus*, *K. sucrofermentans* and *K. pomaceti* [5,6,37]. The BC produced by AAB is considered as generally recognized as safe (GRAS) food by the US Food and Drug Administration (FDA) [42]. To obtain high productivity of BC, the Hestrin-Schramm (HS) medium is favorable. However, its price is quite expensive [43]. Accordingly, the residual agricultural products and alternatives have been selected as substrate for BC cultivation instead of HS medium such as waste beer yeast [43], waste from pineapple, sugar cane juice and rice wine distillery [44,45], and wheat straw hydrolysates [46]. The environmental and nutritional parameters on BC production e.g., temperature, pH, carbon and nitrogen sources were reported by several researchers [47,48,49,50,51]. In current study, the optimum temperature on BC production was 20–30 °C which were not different from the other reports. The temperature is considered to play a critical role in the production of BC. Previous studies indicated that *K. xylinus* SB3.1 can produce BC at the temperature of 15–35 °C but not for 40 °C [52], while *Acetobacter pasteurianus* RSV-4 (MTCC 25117) shows the highest BC production at 30 °C [53]. On the other hand, *K. maltaceti* P285, *K. pomaceti* O277 and *K. nataicola* TISTR 975 were unable to produce BC at the temperature above 35 °C. A low BC production under high incubation temperature has been described that may be due to the denaturation of bacterial cell components e.g., nucleic acids and proteins [54]. The lag phase was not found in the cultivation of *K. xylinus* 0461 at 25–30 °C, whereas a more extended period of log phase was exhibited at the temperature below 20 °C and above 35 °C [55]. Meanwhile, the influence of initial media pH on BC production depends on bacterial strain. Remarkably, *K. maltaceti* P285 and *K. pomaceti* O277 elicited the highest BC production with initial media pH 9.0 and 10.0, respectively which significantly different from the reference strain *K. nataicola* TISTR 975 (optimal pH 6.0) and most reports. Nevertheless, some reports revealed production ability of BC under alkaline condition. For example, *K. intermedius* FST213-1 can synthesize BC at pH 4–9 and demonstrates the highest BC production at pH 8 [56]. The production of BC under alkaline condition has been described to associate with the protective material ability of BC on bacterial cells from extreme pH [56]. It has normally been known that bacterial acid production leads to the reduction of environmental pH and growth inhibition of competitors [57,58,59]. The utilization of glucose as a carbon source for BC production causes the accumulation of acetic acid, gluconic acid and lactic acid in static cultures leading to a reduction of media pH [4,54]. In addition, the suitable media pH is significant for oxidative reaction, cellular nutrient uptake, enzyme function and BC production rate of cellulose-producing bacteria [60].

Basically, glucose will be transformed to glucose-6-phosphate by phosphorylation with glucokinase and then isomerization into glucose-1-phosphate by phosphoglucomutase. The uridine diphosphate (UDP)-glucose is subsequent generated by glucose-1-phosphate uridylyltransferase [61,62]. Ultimately, BC is synthesized using cellulose synthase complexes at the plasma membrane from (UDP)-glucose [61]. The previous study by Son et al. [63] suggested that glucose is mainly consumed in producing BC which consistent with the highest BC production when glucose (16%, *w*/*v*) and sucrose (32%, *w*/*v*) are applied as carbon source. Besides, the influence of complex nitrogen sources illustrated a rising of BC production when the concentration was higher. Conversely, BC production was decreased when the concentration of inorganic nitrogen source was higher which corresponded to the study by Chai and Adnan [64]. The excess nitrogen source can inhibit microbial cellulose synthesis by disruption cell metabolism leading to a loss of nutrient absorption ability. Additionally, the present study indicated that sugarcane and honey are great substrate for BC production (better than HS medium). Sugarcane has been reported to be rich in sugars (glucose, fructose and sucrose), amino acids and organic acids [18,19]. Similarly, honey mainly comprises of sugars and several nutrients e.g., proteins, amino acids, organic acids, vitamins and minerals [26]. Accordingly, *K. maltaceti* P285 isolated from contaminated honey wine has similar BC production potential to a commercial strain, *K. pomaceti* O277. Due to its source of isolation, *K. maltaceti* P285 is highly possible to ferment honey and produce BC. Noteworthy, the honey factories produce several goods from honey and there are plenty honey residues in the containers. This residual honey can be used to produce BC in order to add value and reduce waste. Some sugarcane industries experience the similar occurrence. BC production can be one of the great ways for making more profits on these agricultural products and the strain *K. maltaceti* P285 is promising due to its high favorable pH which can lower cost of pH control in large scale fermentation process.

The characterization of BC produced by *K. maltaceti* P285 grown in HS medium was undertaken using SEM, FTIR, X-ray diffraction, DSC and tensile strength analysis. The SEM analysis of BC was achieved on both surface and cross section. The surface of the BC membrane was reported to contain the microporous (0.1–2 µm) and nano-networks structures (~50–100 nm) [65]. The FTIR spectrum of BC produced by *K. maltaceti* P285 expressed the patterns of characteristic absorption corresponding to the study of Trilokesh and Uppuluri [41]. The −OH and C-H groups stretching was observed at the peaks of 3347–3450 [66] and 2897–2900 cm^−1^ [67], respectively. Similarity, the absorption spectra band at 1639 cm^−1^ is assigned as the hydroxyl group bending [68]. Meanwhile, the peaks at 1462–1481 cm^−1^ corresponded to CH_2_ bending [69]. The absorption band at 1387 and 1049 cm^−1^ exhibits the OH bending and C-O-C pyranose ring skeletal vibration, respectively [70]. The crystallinity BC characterization was carried out by X-ray diffraction analysis. The previous study by Rosa et al. [67] indicated that the peak at around 2θ = 22° is a main crystalline peak, which confirms the appearance of crystalline cellulose. In this work, the major crystalline peak was observed at 22.640°. The DSC analysis of BC presented two peaks at 104 and 341 °C. The peaks between room temperature and 200 °C have been assigned as evaluation of water and solvent [71]. The peak at temperature higher than 300 °C is observed as a decomposition of BC membranes [72]. The degradation of BC material is affected from structural variables e.g., molecular mass, crystallinity and fibers arrangement [73]. Additionally, the mechanical property of BC was tested by tensile assay. The results indicated that the hydrated BC had Young’s modulus and elongation at break values higher than that of dried BC. This phenomenon has been reported by Perez-Rigueiro et al. [74] that related with water molecules incorporated in BC structures, which serves as a plasticizer. The immersion in water of BC interrupts the presence of the hydrogen bonds between molecules. This leads to softening of the structure and increase of ductility [75].

## 5. Conclusions

*K. maltaceti* P285 isolated from contaminated honey wine had a high potential for BC production. The optimal conditions for BC production were 20–30 °C, initial media pH of 9, 16% (*w*/*v*) glucose and 3.2% (*w*/*v*) yeast extract. Higher BC production was obtained when grown in sugarcane or honey solutions. The BC pellicles produced by *K. maltaceti* P285 had similar physical properties to the other reported BC producing strains. *K. maltaceti* P285 is a promising BC producer using residual agricultural and factory products or wastes with alkaline conditions.

## Figures and Tables

**Table 1:** The composition of new formulas of sugarcane (S1 and S2) and honey (H1 and H2) solutions.

Component	CS	S1	S2	CH	H1	H2
Sugarcane (8%, *w*/*v*)	+	+	+	−	−	−
Honey (1:4 of honey: water ratio)	−	−	−	+	+	+
Yeast extract (0.2%, *w*/*v*)	+	−	−	+	−	−
Yeast extract (3.2%, *w*/*v*)	−	+	+	−	+	+
pH 5.0	−	+	−	−	+	−
pH 6.0	+	−	−	+	−	−
pH 9.0	−	−	+	−	−	+

The symbol “+” and “−” displayed the with and without substance addition, respectively. “CS” and “CH” showed the composition of sugarcane and honey solution controls, respectively.

**Table 2:** Biochemical characteristics of isolated bacterial strains P285 and O277, and *K. nataicola* TISTR 975.

Test	Strain P285	Strain O277	*K. nataicola* TISTR 975
Catalase	+	+	+
Citrate	−	+	+
Cytochrome oxidase	−	−	−
Gelatinase	−	−	−
Indole	−	−	−
Lysine deaminase	−	−	−
Lysine decarboxylase	+	+	+
Methyl red	+	+	−
Nitrate reduction	−	−	−
String	+	+	+
Voges-Proskauer	−	−	−
Urease	−	−	−
Carbohydrate fermentation			
Glucose	+	+	w
Galactose	+	+	w
Mannitol	+	w	+
Mannose	w	−	−
Lactose	w	−	−
Maltose	w	w	w
Rhamnose	w	−	−
Sucrose	w	w	w

The symbols “+” and “−” expressed positive and negative results, respectively. The symbol “w” presented weak acid production.

## Data Availability

All data underlying the results are included as part of the published article.

## Supplementary Materials

The following supporting information can be downloaded at: https://www.mdpi.com/article/10.3390/microorganisms10030528/s1, Figure S1: Final media pH of HS broth cultivated by *K. maltaceti* P285, *K. pomaceti* O277 and *K. nataicola* TISTR 975 and incubated at different temperatures for 7 days (A). (B) Final media pH of culture broth after cultivation in HS broth with varying initial media pH and incubated at 30 °C for 7 days., Figure S2: Final media pH of modified MSM with varying carbon sources; citric acid (A), ethanol (B), glucose (C) and sucrose (D) cultivated by *K. maltaceti* P285, *K. pomaceti* O277 and *K. nataicola* TISTR 975 and incubated at 30 °C for 7 days., Figure S3: Final media pH of modified MSM with different nitrogen sources; peptone (A), yeast extract (B), (NH_4_)_2_HPO_4_ (C) and NaNO_3_ (D) cultivated by *K. maltaceti* P285, *K. pomaceti* O277 and *K. nataicola* TISTR 975 and incubated at 30 °C for 7 days., Figure S4: Final media pH of sugarcane (A) and honey (B) solutions when cultivated by *K. maltaceti* P285, *K. pomaceti* O277 and *K. nataicola* TISTR 975 and incubated at 30 °C for 7 days., Figure S5. Final media pH of modified sugarcane (S1 and S2) and honey (H1 and H2) solutions when cultivated by *K. maltaceti* P285, *K. pomaceti* O277 and *K. nataicola* TISTR 975 and incubated at 30 °C for 7 days. The HS broth and the solutions of sugarcane (8%, *w*/*v*; CS) and honey (1:4 of honey: water ratio; CH) supplemented with 0.2% (*w*/*v*) yeast extract, pH 6.0 were used as controls., Table S1: Dry mass of bacterial cellulose produced by *K. maltaceti* P285 and *K. pomaceti* O277 when cultured in modified MSM with different concentrations of carbon and nitrogen sources The *K. nataicola* TISTR 975 was used as a control. Data expressed mean ± standard deviation of three independent experiments. The difference letters were considered statistically significant (*p* < 0.05).
