# Supplementary material for: Characterization of Bacterial Cellulose Produced by Komagataeibacter maltaceti P285 Isolated from Contaminated Honey Wine
Source: Microorganisms. 2022 Feb 28;10(3):528. doi: 10.3390/microorganisms10030528 (PMC8955979; doi:10.3390/microorganisms10030528)
Supplement: Supplementary file 1 [file microorganisms-10-00528-s001.zip › microorganisms-1598511-supplementary.pdf]

Supplementary Material

Characterization of Bacterial Cellulose Produced by *Komagataeibacter maltaceti* P285 Isolated from Contaminated Honey Wine

Narumol Thongwai, Wirapong Futui, Nanthiwa Ladpala, Benjamat Sirichai, Anuwat Weechan, Jirapat Kanklai and Patthanasak Rungsirivanich

Legends of Supplementary Figures and Tables

Supplementary Figure S1. Final media pH of HS broth cultivated by *K. maltaceti* P285, *K. pomaceti* O277 and *K. nataicola* TISTR 975 and incubated at different temperatures for 7 days (A). (B) Final media pH of culture broth after cultivation in HS broth with varying initial media pH and incubated at 30°C for 7 days.

Supplementary Figure S2. Final media pH of modified MSM with varying carbon sources; citric acid (A), ethanol (B), glucose (C) and sucrose (D) cultivated by *K. maltaceti* P285, *K. pomaceti* O277 and *K. nataicola* TISTR 975 and incubated at 30°C for 7 days.

Supplementary Figure S3. Final media pH of modified MSM with different nitrogen sources; peptone (A), yeast extract (B), $(NH_4)_2HPO_4$ (C) and $NaNO_3$ (D) cultivated by *K. maltaceti* P285, *K. pomaceti* O277 and *K. nataicola* TISTR 975 and incubated at 30°C for 7 days.

Supplementary Figure S4. Final media pH of sugarcane (A) and honey (B) solutions when cultivated by *K. maltaceti* P285, *K. pomaceti* O277 and *K. nataicola* TISTR 975 and incubated at 30°C for 7 days.

Supplementary Figure S5. Final media pH of modified sugarcane (S1 and S2) and honey (H1 and H2) solutions when cultivated by *K. maltaceti* P285, *K. pomaceti* O277 and *K. nataicola* TISTR 975 and incubated at 30°C for 7 days. The HS broth and the solutions of sugarcane (8%, *w/v*; CS) and honey (1:4 of honey : water ratio; CH) supplemented with 0.2% (*w/v*) yeast extract, pH 6.0 were used as controls.

Supplementary Table S1. Dry mass of bacterial cellulose produced by *K. maltaceti* P285 and *K. pomaceti* O277 when cultured in modified MSM with different concentrations of carbon and nitrogen sources The *K. nataicola* TISTR 975 was used as a control. Data expressed mean ± standard deviation of three independent experiments. The difference letters were considered statistically significant ($p < 0.05$).

Supplementary Figure S1

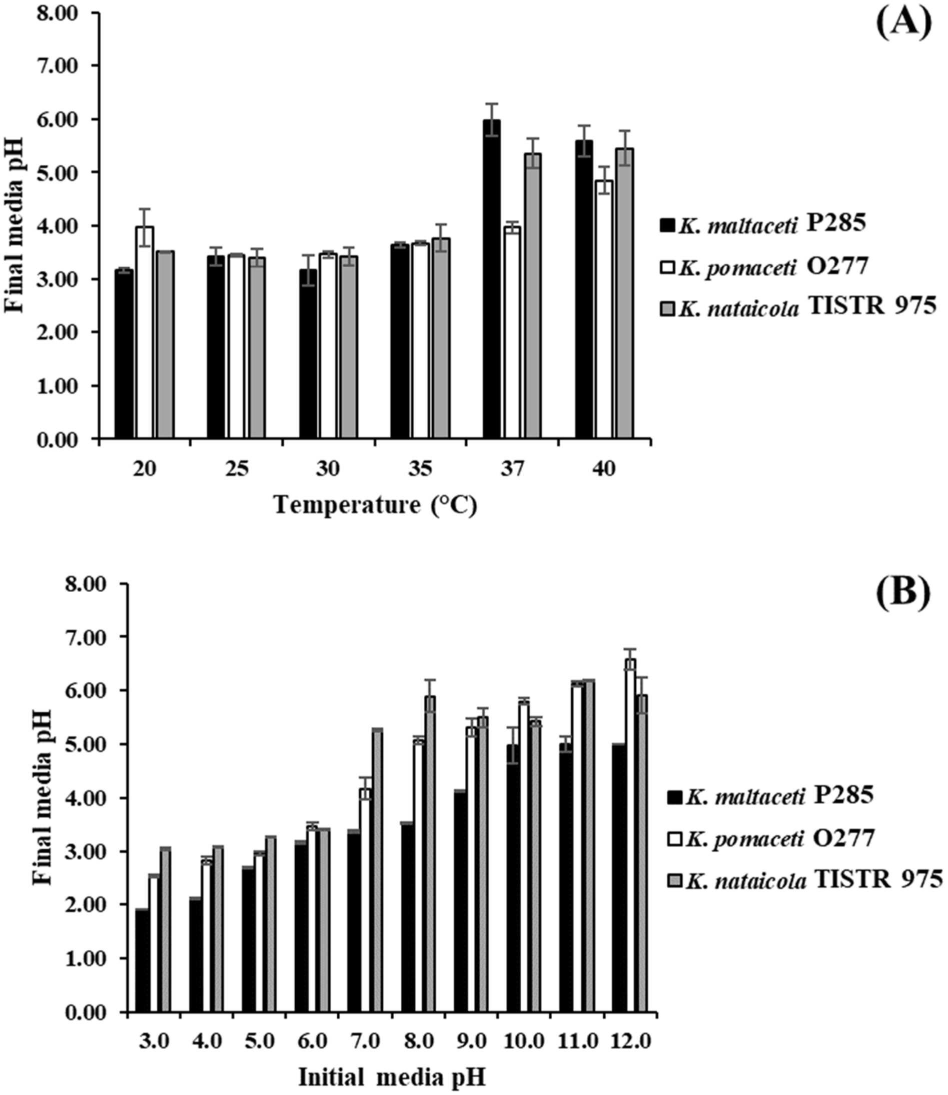

Supplementary Figure S2

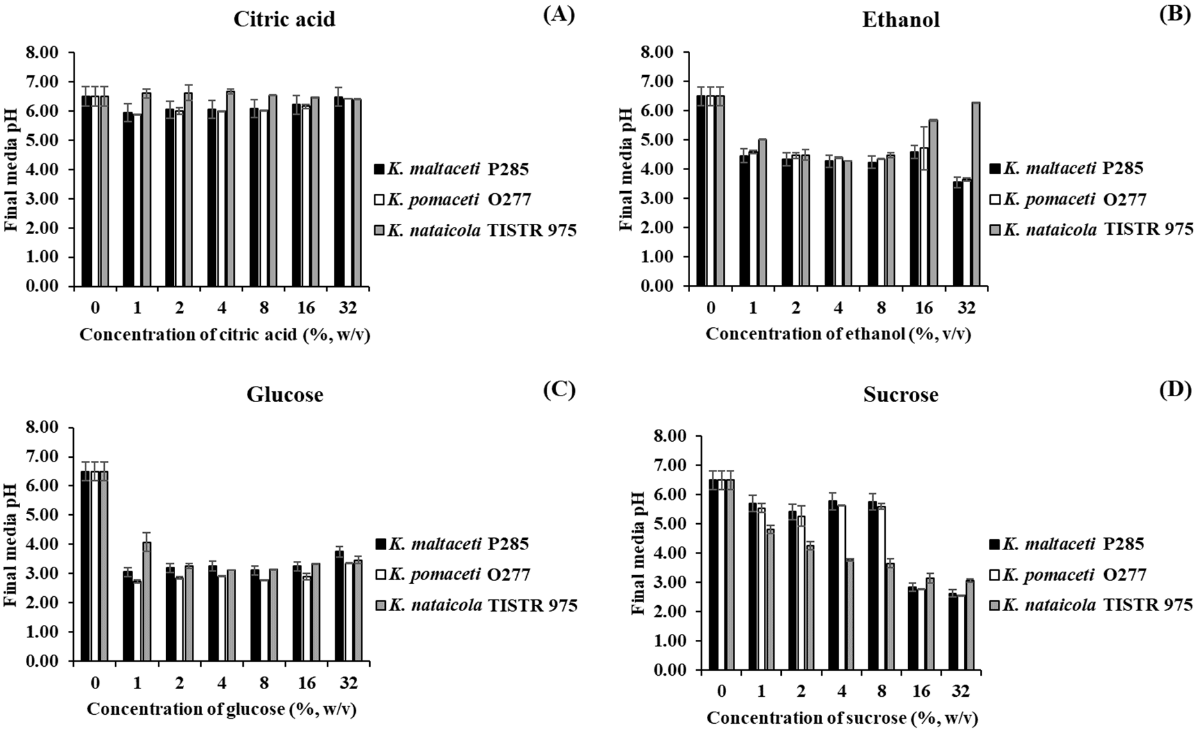

Supplementary Figure S3

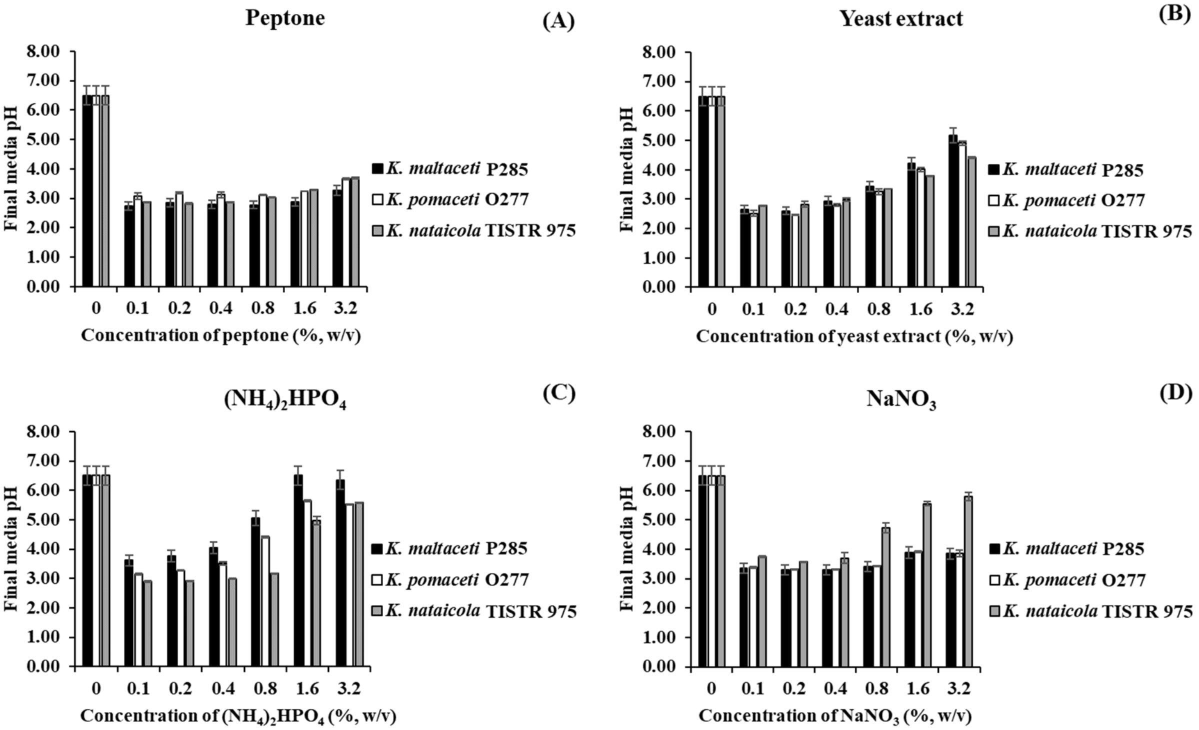

Supplementary Figure S4

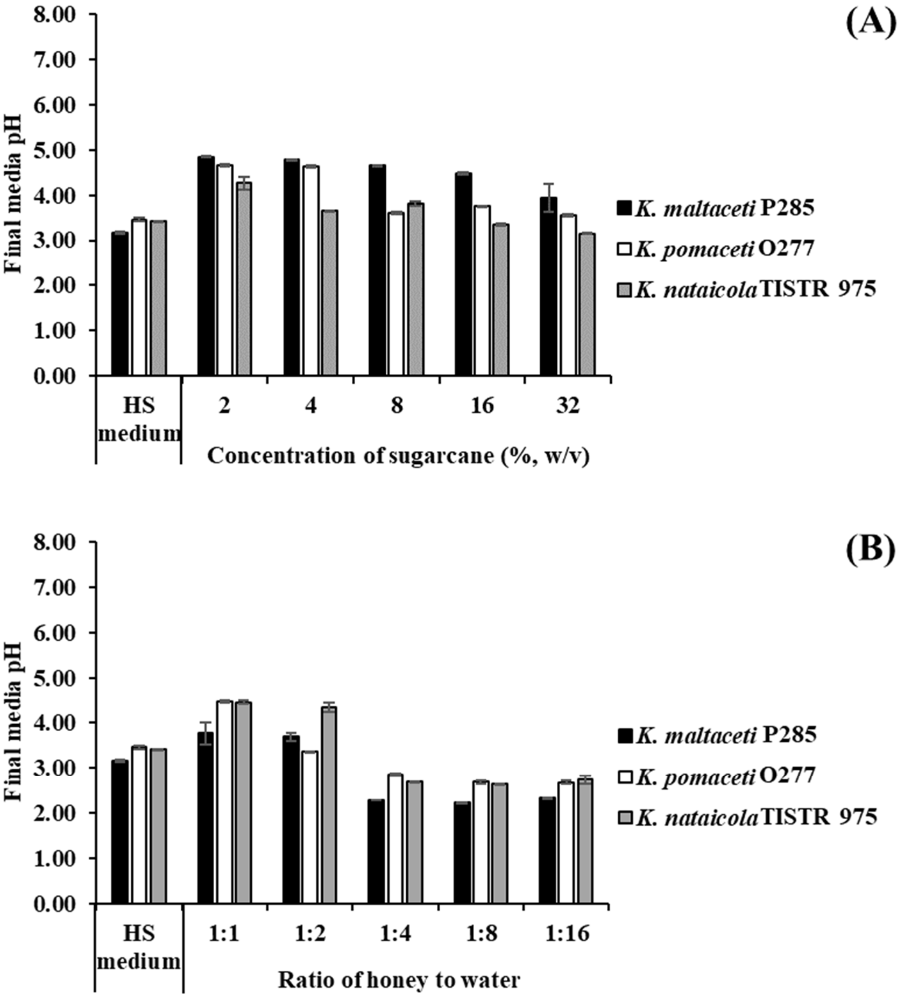

Supplementary Figure S5

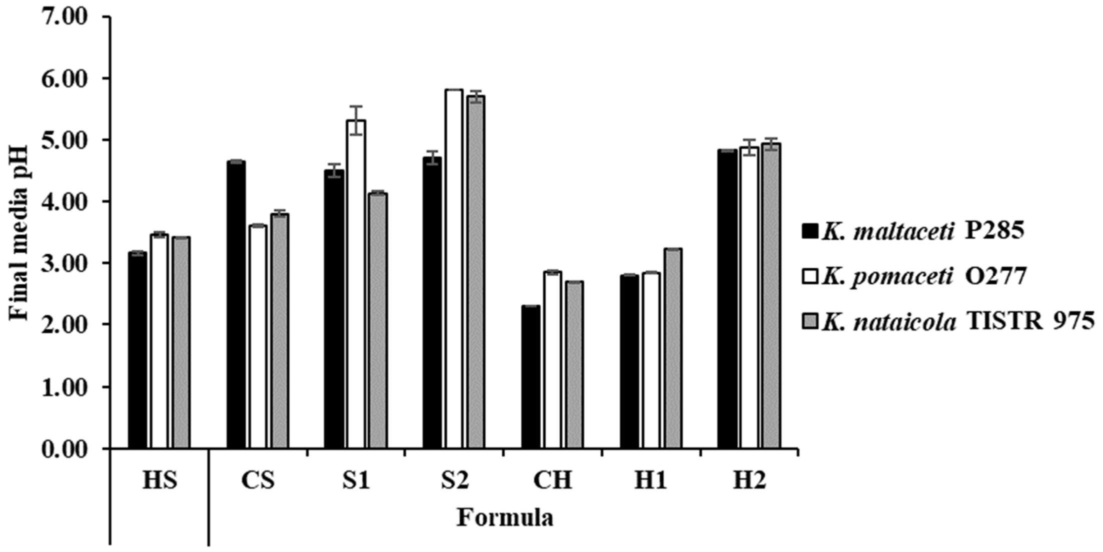

Supplementary Table S1

	Dry weight of cellulose (g/L)											
Carbon source concentration	*Komagataeibacter maltaceti* P285				*Komagataeibacter pomaceti* O277				*Komagataeibacter nataicola* TISTR 975			
	Citric acid	Ethanol	Glucose	Sucrose	Citric acid	Ethanol	Glucose	Sucrose	Citric acid	Ethanol	Glucose	Sucrose
0%	0^{a}	0^{a}	0^{f}	0^{g}	0^{a}	0^{a}	0^{f}	0^{g}	0^{d}	0^{a}	0^{f}	0^{g}
1%	0^{a}	0^{a}	1.2±0.1^{e}	0.6±0.0^{f}	0^{a}	0^{a}	1.2±0.2^{e}	1.2±0.1^{f}	0.2±0.1^{c}	0.1±0.0^{a}	2.9±0.0^{e}	0.7±0.1^{f}
2%	0^{a}	0^{a}	3.0±0.1^{d}	1.7±0.1^{e}	0^{a}	0^{a}	3.0±0.1^{d}	3.3±1.1^{e}	0.6±0.1^{b}	0^{a}	7.3±0.1^{d}	1.6±0.2^{e}
4%	0^{a}	0^{a}	4.2±0.2^{c}	3.2±0.2^{d}	0^{a}	0^{a}	4.4±0.9^{c}	6.2±1.4^{d}	0.9±0.1^{a}	0^{a}	15.1±0.6^{c}	2.1±0.1^{d}
8%	0^{a}	0^{a}	7.2±0.4^{b}	4.2±0.2^{c}	0^{a}	0^{a}	8.2±0.4^{b}	8.1±0.7^{c}	0.5±0.2^{b}	0^{a}	20.5±0.6^{b}	5.3±1.2^{c}
16%	0^{a}	0^{a}	11.3±0.6^{a}	4.9±0.2^{bc}	0^{a}	0^{a}	11.9±1.6^{a}	9.4±0.2^{b}	0^{d}	0^{a}	21.7±0.6^{a}	8.0±1.1^{b}
32%	0^{a}	0^{a}	1.2±0.1^{e}	6.7±0.3^{a}	0^{a}	0^{a}	1.2±0.4^{e}	12.8±1.3^{a}	0^{d}	0^{a}	16.0±0.5^{c}	16.7±1.2^{a}
Nitrogen source concentration	*Komagataeibacter maltaceti* P285				*Komagataeibacter pomaceti* O277				*Komagataeibacter nataicola* TISTR 975			
	Peptone	Yeast extract	$(NH_4)_2HPO_4$	$NaNO_3$	Peptone	Yeast extract	$(NH_4)_2HPO_4$	$NaNO_3$	Peptone	Yeast extract	$(NH_4)_2HPO_4$	$NaNO_3$
0%	0^{d}	0^{e}	0^{d}	0^{c}	0^{c}	0^{e}	0^{e}	0^{c}	0^{g}	0^{f}	0^{e}	0^{e}
0.1%	1.6±0.0^{c}	1.3±0.1^{d}	1.3±0.0^{b}	2.8±0.1^{a}	2.0±0.0^{b}	1.0±0.0^{d}	2.9±0.0^{b}	2.8±0.1^{a}	3.9±0.2^{f}	5.9±0.5^{e}	5.0±0.6^{c}	6.5±0.0^{b}
0.2%	1.7±0.2^{c}	1.2±0.0^{d}	1.7±0.1^{a}	3.0±0.1^{a}	2.0±0.2^{b}	1.0±0.0^{d}	3.9±0.9^{a}	3.0±0.0^{a}	5.0±0.3^{e}	6.3±0.3^{e}	3.9±0.1^{d}	7.3±0.5^{a}
0.4%	2.3±0.0^{b}	1.3±0.1^{d}	1.6±0.2^{a}	3.1±0.2^{a}	2.8±0.8^{ab}	1.0±0.2^{d}	3.6±0.0^{a}	3.1±0.2^{a}	6.1±0.2^{d}	7.6±0.8^{d}	6.5±0.5^{b}	7.5±0.1^{a}
0.8%	1.7±0.0^{c}	3.8±0.2^{c}	1.1±0.1^{b}	1.6±0.1^{b}	2.0±0.3^{b}	3.1±0.2^{c}	2.6±0.1^{c}	1.6±0.1^{b}	9.6±1.5^{c}	9.8±0.3^{c}	11.1±0.2^{a}	2.2±0.3^{c}
1.6%	1.7±0.1^{c}	7.1±0.4^{b}	0.9±0.0^{c}	1.5±0.1^{b}	2.1±0.1^{b}	5.7±0.0^{b}	2.0±0.0^{d}	1.6±0.0^{b}	15.6±0.8^{b}	13.0±0.6^{b}	10.7±0.2^{a}	2.1±0.1^{c}
3.2%	2.7±0.1^{a}	11.9±0.6^{a}	0^{d}	0^{c}	3.3±0.3^{a}	9.6±0.1^{a}	0.3±0.0^{e}	0^{c}	18.1±0.5^{a}	21.1±0.2^{a}	0^{e}	0.7±0.0^{d}
